# Amputations in adult burns patients: a 10-year retrospective study

**DOI:** 10.1308/rcsann.2024.0117

**Published:** 2025-04-03

**Authors:** E Mathews, E Chipp

**Affiliations:** ^1^ University of Birmingham Medical School, UK; ^2^ University Hospitals Birmingham NHS Foundation Trust, UK

**Keywords:** Burns, Amputation, Diabetes, TBSA

## Abstract

**Introduction:**

Amputation is an uncommon but potentially life-changing complication of burns. No studies of amputation among UK burns patients currently exist.

**Methods:**

A 10-year review of burns patients with amputations at the Queen Elizabeth Hospital Birmingham was conducted. Descriptive analysis was undertaken to identify patient characteristics. Statistical analysis was conducted to identify relationships between patient and injury details and the number of amputations, and relationships between the number of amputations and patient outcomes.

**Results:**

Thirty-five adult burns patients (mean age 48.1 years, 65.7% male) were identified, 62.9% of whom suffered flame burns. The median total body surface area (TBSA) burned was 24%. The amputation risk among admitted burns patients was 1.2%. Major burns patients (≥25% TBSA burned) underwent more minor (*p*=0.018) and upper limb amputations (*p*=0.035) compared with minor burns patients. Median length of hospital stay was 67.5 days. Length of stay was positively correlated with the number of total (*p*=0.001), minor (*p*=0.004) and upper limb (*p*=0.002) amputations. In total, 67.6% of amputees underwent revisional procedures. The number of revisions was positively correlated with the number of major (*p*=0.010) and lower limb (*p*=0.001) amputations.

**Conclusions:**

A minority of adult burns patients underwent amputations. Patient and injury information may predict a greater number of amputations, which in turn may predict longer hospital stays and a requirement for more revisional procedures. This information could be used to better counsel patients about their likely outcomes. A multicentre case-control study is required to clarify risk factors for amputation in burns.

## Introduction

Amputation is required in burns patients when salvage of the affected body part is impossible because of non-viable tissue, lack of blood supply, infection or severe scar contractures.^1^ Removing necrotic tissue reduces the systemic response to a burn and reduces infection risk.^2^ It has been suggested that amputation in burns patients reduces morbidity and increases survival.^3–5^

Amputation results in multiple physical and psychosocial issues. Studies show that 95% of amputees experience phantom limb pain, residual limb pain or back pain, 71.2% experience major depressive disorder, 30.5% experience suicidal ideations and 20.3% experience post-traumatic stress disorder.^6,7^ Amputees often feel excluded, lose their independence and are unable to resume normal daily activities and work.^8^ This study may aid healthcare practitioners in identifying patients more likely to require amputations following a burn, enabling intervention for the preservation of the burned body part, or preparation for patient discussions about the requirement for amputation, which may improve physical and psychosocial recovery.

Limited literature exists regarding amputation following burn injury, and the majority of available studies consider only amputation following electrical burns. There are currently no United Kingdom (UK) studies of amputations in adult burns patients. This study fills this gap, identifying the first amputation risk among UK burns patients, factors that result in more amputations and factors that alter postamputation outcomes.

The aims of this study are listed in Table 1.

**Table 1:** Aims of this study

1	Identify amputation incidence among adult burns patients and describe amputee demographics, comorbidities, burn information and outcomes.
2	Analyse the relationship between patient demographics, comorbidities and burn-specific factors with the number of amputations required.
3	Analyse the relationship between the number of amputations that patients require and their postamputation outcomes.

## Methods

This single-centre retrospective study reviewed patients who underwent amputation owing to a burn injury between 1 September 2012 and 1 September 2022. This project is registered with the Queen Elizabeth Hospital Birmingham (QEHB) Clinical Audit Registration and Management System with the audit code CARMS-18675 and was run as a service evaluation. Ethical review was therefore not required given the retrospective and non-generalisable nature of this work. This study is reported according to the Strengthening the Reporting of Observational Studies in Epidemiology (STROBE) guidelines.^9^

All UK burns data are recorded in the International Burns Injury Database.^10^ Interrogation of this database produced a list of all QEHB burns patients who underwent an operative procedure between 1 September 2012 and 1 September 2022. Adult patients (≥16 years) who underwent an amputation secondary to burns, including those with polytrauma in whom the body part was amputated owing to a burn injury, at the QEHB between September 2012 and September 2022 were included. Those who underwent primary amputation elsewhere or whose amputation was not because of a burn injury, including non-burn skin loss (e.g. purpura fulminans, toxic epidermal necrolysis), were excluded. Patient data were collected from the QEHB electronic patient records systems without identifiable information.

The data collection variables are detailed in Appendix A (available online).

### Statistical analysis

Throughout the analysis, the normality of the distribution of continuous variables was tested for with the Shapiro–Wilk test. A *p*-value <0.05 was considered statistically significant. Statistical analysis was performed using Statistical Package for the Social Sciences (SPSS) version 29. Patients were excluded from the analysis of any variable for which they had missing data.

Amputation risk was calculated by dividing the number of burns amputees by the total number of acute burns admissions. Categorical variables were summarised as frequencies and percentages, and continuous data as mean and standard deviation (sd) or median and interquartile range (IQR), depending on normality.

The number of total, major (proximal to or at the wrist or ankle), minor (distal to the wrist or ankle), upper limb and lower limb amputations that patients underwent was compared for the following groups: male vs female; presence or absence of a medical condition (diabetes mellitus, myocardial infarction or angina, transient ischaemic attack [TIA] or stroke, hypertension, atrial fibrillation, deep vein thrombosis [DVT] or pulmonary embolism, heart failure [HF], peripheral vascular disease [PVD], epilepsy); smokers vs non-smokers; intravenous drug users (IVDU) vs non-IVDU; medicated patients vs non-medicated patients (warfarin, novel oral anticoagulants [NOACs] and steroids); and major burns (≥25% total body surface area [TBSA] burned) vs minor burns (<25% TBSA burned).

The Student’s *t*-test was used where the dependent variable was normally distributed in both comparator groups, and the Mann–Whitney *U* test was used where it was not.

The number of total, major, minor, upper limb and lower limb amputations that patients underwent was also compared between ethnicities, causes of burn, mechanisms of burn, anatomical locations of burn and burn depths, using one-way analysis of variance for normally distributed data and the Kruskal–Wallis test for non-normally distributed data.

Finally, correlation between the number of total, major, minor, upper limb and lower limb amputations and age, body mass index (BMI), %TBSA burned and peak creatine kinase (CK) was assessed, using Pearson’s correlation coefficient for normally distributed data and Spearman’s rank correlation coefficient for non-normally distributed data.

Using the methods described above, the number of total, major, minor, upper limb and lower limb amputations that patients underwent were compared between the following groups using either the Student’s *t*-test or the Mann–Whitney *U* test: presence or absence of wound infection; wound dehiscence; mortality; and patients who were discharged home vs those who were discharged elsewhere.

Correlation was analysed between the number of total, major, minor, upper limb and lower limb amputations, and length of hospital stay and number of revisional procedures required.

## Results

**Figure 1:**
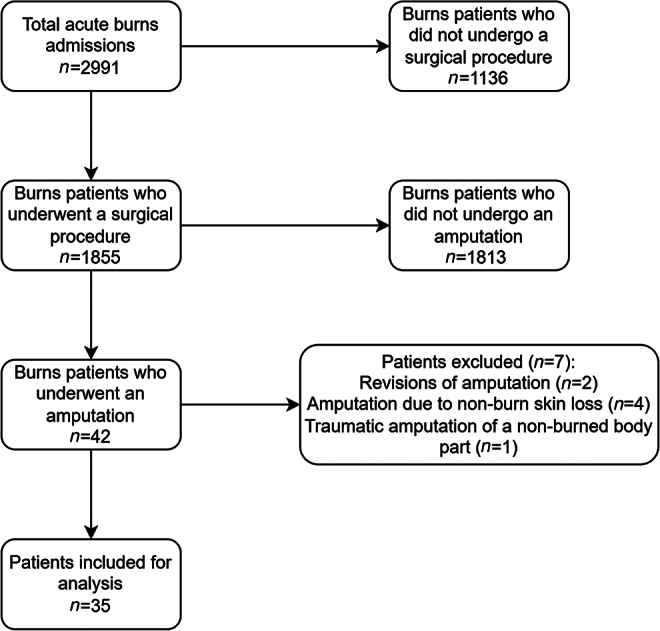
Selection process of patients for analysis, leaving 35 included

Between 1 September 2012 and 1 September 2022, there were 2,991 acute admissions to the QEHB burns centre. Following application of our inclusion and exclusion criteria, 35 patients remained for analysis (Figure 1). None had amputations prior to their burn injury.

### Amputation risk

The amputation risk among adult burns patients at the QEHB was 1.2% (35 amputees of 2,991 acute burns admissions).

### Patient demographics

Patient demographics are reported in Table 2. No differences in the number of total, major, minor, upper limb or lower limb amputations were identified between genders or ethnicities. There was no correlation between age and the number or type of amputations (Appendix B – available online).

**Table 2:** Patient demographics

Characteristic	Results
Age (years)*	48.1 (17.9)
Gender	
Male	23 (65.7)
Female	12 (34.3)
Ethnicity	
White British	21 (67.7)
Asian or Asian British – Indian	3 (9.7)
Asian or Asian British – Pakistani	2 (6.5)
White – any other white background	4 (12.9)
Black/Black British – Caribbean	1 (3.2)

Data are given as number (%), except * mean (sd). Age and gender data were available for all patients. Ethnicity data were available for 34 patients

### Past medical and drug history

Patient BMI and past medical and drug history are reported in Table 3. No differences in the number of total, major, minor, upper limb or lower limb amputations were identified between patients with vs without diabetes, hypertension or epilepsy. There was also no correlation between BMI and the number of total, major, minor, upper limb or lower limb amputations (Appendix C – available online). Smoking status was available for 12 patients, 8 of whom were smokers. No patients had recorded cases of TIA/stroke, DVT, HF, PVD, IVDU or NOAC use.

**Table 3:** Past medical and drug histories of the amputees

Medical condition or drug	Result
Body mass index (kg/m^2^)*	24.5 (6.3)
Hypertension	9 (26.5)
Diabetes mellitus	6 (17.6)
Epilepsy	3 (8.8)
Myocardial infarction/angina	2 (5.9)
Atrial fibrillation	2 (5.9)
Pulmonary embolism	1 (2.9)
Warfarin	1 (2.9)
Steroids	1 (2.9)

Data are given as number (%), except * mean (sd)

Body mass index and past medical and drug history data were only available for 34 patients

### Burn-specific information

Burn-specific information is recorded in Table 4. No differences in the number of total, major, minor, upper limb or lower limb amputations were identified between the different causes of burn or mechanisms of injury (Appendix D – available online).

**Table 4:** Burn-specific information

Variable	Result
Cause of burn	
Flame	22 (62.9)
Scald	4 (11.4)
Contact	5 (14.3)
Electrical	2 (5.7)
Chemical	2 (5.7)
Mechanism of injury	
Home accident	16 (45.7)
Workplace injury	5 (14.3)
DSH	5 (14.3)
Epileptic seizure	3 (8.6)
RTA	1 (2.9)
Other	5 (14.3)
Anatomical location of burn	
Multiple sites	22 (64.7)
Upper limb	6 (17.6)
Lower limb	6 (17.6)
%TBSA burned*	24 (52.5)
Extent of burn	
Major (≥25% TBSA)	17 (50)
Minor (<25% TBSA)	17 (50)
Burn depth	
Mixed	15 (45.5)
Full thickness	18 (54.5)
Peak creatine kinase (ng/ml)*	925 (8610)
Escharotomy	16 (47.1)
Fasciotomy	6 (17.6)
Fluid input (ml/kg/%TBSA burned)†	5.1 (0.96)

DSH = deliberate self-harm; RTA = road traffic accident; TBSA = total body surface area

Data are given as number (%), except *median (IQR) and †mean (sd). Data were available for all 35 patients for burn cause and mechanism of injury, 34 for anatomical location of burn, 33 for burn depth, 21 for peak CK level, and 20 for fluid input

No correlations were identified between the %TBSA burned and number of total, major, minor, upper limb or lower limb amputations (Appendix D – available online). However, major (≥25% TBSA) burns patients underwent significantly more minor amputations (median 2, IQR 4) than minor burns patients (median 1, IQR 2) with a *p*-value of 0.018. Major burns patients also underwent more upper limb amputations (median 2, IQR 4) than minor burns patients (median 1, IQR 1.5), with a *p*-value of 0.035.

There were no correlations between peak CK and the number of total, major, minor, upper limb or lower limb amputations (Appendix D – available online).

In total, 15 patients had burns <15% TBSA, and therefore did not require fluid resuscitation (the QEHB uses 15% TBSA as a cut-off for initiating resuscitation fluids). The fluid input for the remaining 20 patients is recorded in Table 4.

### Amputation-specific information

Amputation-specific information is reported in Table 5. Of 22 patients who had upper limb amputations, 18 (81.8%) had minor and 4 (18.2%) had major amputations. Of ten patients who had lower limb amputations, three (30%) had minor and seven (70%) had major amputations. Three patients had both major and minor or upper limb and lower limb amputations, and so were not included here.

**Table 5:** Amputation-specific information

Variable	Result
Timing to first or only amputation (days post burn)*	28 (50)
Total amputations per patient*	2 (3)
Major amputations per patient*	0 (1)
Minor amputations per patient*	1 (4)
Upper limb amputations per patient*	1 (4)
Lower limb amputations per patient*	0 (1)
Level of amputation	
Distal to PIPJ	52 (55.3)
Proximal to PIPJ	16 (17.0)
Below elbow	2 (2.1)
Above elbow	3 (3.2)
Below knee	3 (3.2)
Through knee	1 (1.1)
Above knee	7 (7.4)
Through foot	1 (1.1)
Toe	9 (9.6)
Reason for amputation	
Non-viable tissue	28 (80)
Infection	2 (5.7)
Iatrogenic	1 (2.9)
Embolus	1 (2.9)
Scar contractures	2 (5.7)
Polytrauma	1 (2.9)
Grade and speciality of surgeon	
Consultant burns surgeon	31 (88.6)
Consultant plastic surgeon	1 (2.9)
Consultant T&O surgeon	1 (2.9)
Consultant vascular surgeon	1 (2.9)
Plastics and Burns Registrar	1 (2.9)

PIPJ = proximal interphalangeal joint; T&O = trauma and orthopaedic

Data are given as number (%), except *median (IQR). Data were available for all 35 patients for timing of amputation, number of amputations, level of amputation, grade and speciality of surgeon, and 33 patients for reason for amputation

### Outcomes following amputation

Outcomes following amputation are recorded in Table 6. There was a statistically significant positive correlation between length of hospital stay and the total number of amputations (*r*=0.538, *p*=0.001), the number of minor amputations (*r*=0.438, *p*=0.004) and the number of upper limb amputations (*r*=0.511, *p*=0.002). Numbers of major and lower limb amputations were not significantly correlated with length of hospital stay (Appendix E – available online).

**Table 6:** Outcomes following amputation

Outcome	Result
Length of hospital stay (days)*	67.5 (87.3)
Discharge destination	
Home	20 (58.8)
Rehabilitation hospital	8 (23.5)
Burns unit	3 (8.8)
Burns facility	1 (2.9)
Inpatient psychiatry unit	1 (2.9)
Women’s refuge	1 (2.9)
Revisional procedures to amputation stump*	1 (2)

Data are given as number (%), except *median (IQR). Data were available for 34 patients for length of hospital stay, discharge destination and number of revisional procedures required

Thirty-four patients survived to discharge; unfortunately, one patient, a 57-year-old with 20% TBSA full-thickness burns and bilateral below-knee amputations, did not survive owing to multiorgan failure.

No differences were found in the number of total, major, minor, upper limb or lower limb amputations between patients discharged home versus those discharged elsewhere (Appendix E – available online).

Twenty-three patients (67.6%) underwent amputation stump revisions. There was a statistically significant positive correlation between the number of major amputations (*r*=0.435, *p*=0.010) and the number of lower limb amputations (*r*=0.537, *p*=0.001) with the number of revisional procedures (Appendix E – available online).

Ten patients experienced chronic pain following their amputation, and five patients, all with major amputations, were seen by prosthetic-fitting services; however, both of these were poorly recorded.

## Discussion

### Amputation risk

This study found a lower amputation risk (1.2%) than previous literature (1.4%–6.7%), possibly because burns care is more standardised in the UK, owing to the presence of a burns network and national burn care standards.^11–16^ In addition, transfer times to specialist burn services are inevitably shorter in the UK than in larger countries with no burns network. Another possible explanation is the low UK rate of electrical injuries, known to be associated with high risk of amputation.^17–19^

Unfortunately, data on transfer of patients to the QEHB were not available. A systematic review of burn care outside burns centres demonstrated that patients were often delivered too much fluid or the wrong fluid type, that wound care was variable and that hypothermia was not managed satisfactorily.^20^ These could all be risk factors for amputation and therefore whether patients were brought directly to the QEHB or came via another facility may have affected the amputation rate.

### Patient demographics

The male majority in this study (65.7%) is similar to national burns data (63% male), suggesting that gender may not be an amputation risk factor in UK burns patients. National age and ethnicity data for burns patients are not available for comparison.^17^

Previous literature linked increased age, male gender and Black ethnicity to increased likelihood of amputation. The lack of a relationship in our study is therefore unexpected, and likely due to the small sample size.^11–13,18,19^

### Past medical and drug history

The impact of BMI on amputations in burns has not been explored in previous literature, and our inconclusive findings necessitate further research.

Previous literature found that burns amputees had a higher Charleston Comorbidity Index than non-amputees.^13^ Numerous studies have found that people with diabetes are significantly more likely to require lower limb amputations than people without diabetes, owing to peripheral neuropathy causing reduced sensation and therefore lack of awareness of a burning process.^21–24^ In addition, epilepsy is a suggested risk factor owing to burns sustained during seizures.^15,25^ However, no relationships between comorbidities and amputations were observed in our study, possibly because of limited patient numbers. The effects of other comorbidities and drug history details were not analysed because of their very low prevalence in this group.

### Burn-specific information

Flame burns constituted 62.9% of injuries seen and electrical burns constituted 5.7%, similar to some previous literature.^13,15^ This may reflect the low prevalence of electrical burns in England and Wales.^17^ However, most previous literature found electrical burns to be the most common cause of amputation because of the greater extent of associated tissue damage.^18,19,26,27^

Contrary to previous literature, no significant differences in amputation numbers were identified between different causes of burn or mechanisms of injury, possibly because of the small sample size.^18,19,26,27^ Mechanism of injury is likely less relevant as a prognosticator for amputation compared with other factors, such as %TBSA burned and burn depth.

Patients most commonly experienced full-thickness burns across multiple sites. This is consistent with previous studies linking full-thickness burns to increased amputation risk because of greater tissue damage.^18^ Owing to challenges in categorising burns into distinct depth levels (especially with mixed-thickness burns) or distinct anatomical areas, comparisons between amputation numbers for different burn depths and anatomical locations were not conducted.

The median %TBSA burned in this study (24%) considerably exceeded the national median for admitted burns patients (2%).^17^ A higher %TBSA burned also indicates a greater extent of tissue damage, potentially increasing the likelihood of amputation. However, no statistically significant correlation between %TBSA burned and the number and type of amputations was observed, possibly because of the small sample size.

Major burns patients underwent significantly more minor and upper limb amputations than minor burns patients, reflecting the greater tissue damage and therefore increased amputation risk associated with a higher %TBSA burned, especially of the digits (minor amputations), and the fingers specifically (upper limb amputations). The lack of significant differences in total, major and lower limb amputations between major and minor burns could again be due to the small sample size.

This study, along with work by Schulze *et al*, demonstrates that there are occasions when despite escharotomy, body parts may remain non-salvageable.^28^ Comparisons based on these interventions and fluid resuscitation are invalid, because these are not indicated for all patients.

Patients requiring fluid resuscitation received volumes similar to ideal standards, suggesting that fluid input did not contribute greatly to amputations in this patient population.^29^ Further research is required to investigate whether poor fluid resuscitation is a risk factor for amputation.

Despite the association in previous studies between higher peak CK and increased amputation risk, no significant correlation was found in this study.^30,31^ The limited peak CK data (21 patients) may account for this lack of correlation. Further research is required to understand this relationship.

### Amputation-specific information

This study found that amputation distal to the proximal interphalangeal joint was the most common. Similarly, Wall *et al* found that amputations at the metacarpophalangeal joints were the most common, and Jang *et al* found that finger amputations were the most common.^15,32^ This is an important result, because finger amputations greatly impact hand function and overall quality of life.

This study found a higher proportion of minor amputations (83.0%) compared with the previous literature, possibly because of the experienced team of specialist burns, hand and plastic surgeons at the QEHB, who were able to preserve limbs and therefore only amputate digits.^18,27^ Other centres lacking similar facilities or experience may have resorted to major amputations. However, this variation may not be due to the surgeons, but rather the burn types received by the QEHB.

Some 81.8% of upper limb amputees had minor amputations, a reflection of the high proportion of finger amputations. By contrast, 70% of lower limb amputations were major, possibly owing to more severe burns or a lower priority of digit salvage compared with upper limb digits.

The median time to amputation (28 days) was longer than identified in previous literature (published more than 20 years ago), possibly because of advances in burns care allowing for more assessment time before an amputation decision.^3,4^ Most amputations occurred during the initial inpatient stay, which is unsurprising, given the acute nature of burn injuries.

Amputation was primarily due to tissue non-viability, a well-known complication of burn injuries. In almost all cases, amputations were performed by a consultant burns surgeon, and in a few cases, plastic, trauma and orthopaedic or vascular surgeons aided the burns team in amputations that required specialist input.

### Outcomes following amputation

Burns amputees in this study had a median hospital stay of 67.5 days, contrasting with a 1-day median stay for all burns patients nationally.^17^ This difference is likely due to the severity of the burns seen in burns amputees and their postoperative care requirements. These findings align with previous literature, in which burns amputees have a significantly longer length of hospital stay than non-amputees.^13,19^

The number of total, minor and upper limb amputations showed a significant positive correlation with length of stay, indicating the increased care needs of these patients. However, major and lower limb amputees were often transferred to rehabilitation hospitals or other burns services, explaining their lack of correlation with length of hospital stay.

The majority of amputees (67.6%) underwent revisional procedures. The proportion of amputees requiring revisions has previously only been reported in electrical burns, so comparison with all burn types in this study is invalid.^30,33^

The number of major and lower limb amputations were both positively correlated with number of revisional procedures, likely because major amputations (which included the majority of lower limb amputations) remove a greater amount of tissue and leave a greater area of stump that is subject to postoperative complications. In addition, as perioperative oedema settles, lower limb amputees frequently undergo revisions to fit prosthetic limbs.

Patients with more amputations were expected to be discharged to other healthcare facilities for further care. The lack of a significant difference between the number of amputations in those patients discharged home vs patients discharged elsewhere could again be due to the small sample size.

Incidence of chronic pain was poorly recorded, limiting any valid conclusions. Only one mortality occurred, and this was due to the systemic response to the burn injury that caused multiorgan failure and was therefore not directly attributable to the amputation. As expected, major amputees were the only patients seen by prosthetic-fitting services, because these patients typically require substantial support for their return to activities of daily living, and prostheses for major amputations are well established.

### Study limitations

This study has several limitations. The single-centre design means that the applicability of the findings to other UK burns services is limited. However, this does mean that the care provided, facilities and resources, and management strategies were similar over time for all patients involved. The retrospective nature and reliance on accurate medical record keeping also limit this study. Only 35 patients were included and this small sample size limits the reliability and applicability of the results.

Another limitation is that patient receipt of first aid is not routinely recorded in hospital notes. First aid significantly reduces burn wound depth, and this is likely a factor that would decrease the requirement for amputations.^34^

## Conclusion

In conclusion, this study offers new insights for UK burns services by identifying the first amputation risk among UK burns patients, as well as factors influencing the number of amputations, length of hospital stay and requirement for revisional procedures. These findings could be used to identify patients at greater risk of more amputations, and therefore intervene to either aim for limb preservation or better counsel patients about their likely outcomes. In the future this work may inform further studies or systematic reviews.

## Funding

This work was supported by the Royal College of Surgeons of England’s Intercalated Bachelor of Science Degree in Surgery or Surgical Related Area grant. This research did not receive any other grants from funding agencies in the public, commercial or not-for-profit sectors.

## Author contributions

All authors contributed significantly to this manuscript and approved the final article. EM: conception and design of study, data acquisition, analysis and interpretation of data, writing of manuscript, final approval of version to be submitted. EC: conception and design of study, data acquisition, editing of manuscript, final approval of version to be submitted.

## References

[1] Thornburg DA, Swanson S, Spadafore P *et al.* Burn center patients at risk for upper extremity amputations. *Plast Surg* 2021. **31**: 229–235. 10.1177/22925503211042863 PMC10467439 37654535

[2] McGovern C, Puxty K, Paton L. Major burns: part 2. Anaesthesia, intensive care and pain management. *BJA Educ* 2022; **22**: 138–145. 35531075 10.1016/j.bjae.2022.01.001 PMC9073309

[3] Viscardi P, Polk H. Outcome of amputations in patients with major burns. *Burns* 1995; **21**: 526–530. 8540981 10.1016/0305-4179(95)00036-b

[4] Yowler C, Mozingo D, Ryan J, Pruitt B. Factors contributing to delayed extremity amputation in burn patients. *J Trauma* 1998; **45**: 522–527. 9751544 10.1097/00005373-199809000-00017

[5] Kennedy P, Young W, Deva A, Haertsch P. Burns and amputations: a 24-year experience. *J Burn Care Res* 2006; **27**: 183–188. 16566562 10.1097/01.BCR.0000203492.89591.A1

[6] Ephraim P, Wegener S, MacKenzie E *et al.* Phantom pain, residual limb pain, and back pain in amputees: results of a national survey. *Arch Phys Med Rehabil* 2005; **86**: 1910–1920. 16213230 10.1016/j.apmr.2005.03.031

[7] Sahu A, Gupta R, Sagar S *et al.* A study of psychiatric comorbidity after traumatic limb amputation: a neglected entity. *Ind Psychiatry J* 2017; **26**: 228–233. 30089974 10.4103/ipj.ipj_80_16 PMC6058428

[8] Behera P, Dash M. Life after lower limb amputation: a meta- aggregative systemic review of the effect of amputation on amputees. *J Disabil Stud* 2021; **7**: 90–96.

[9] Vandenbroucke JP, von Elm E, Altman DG *et al.* Strengthening the Reporting of Observational Studies in Epidemiology (STROBE): explanation and elaboration. *PLoS Med* 2007; **4**: e297. 17941715 10.1371/journal.pmed.0040297 PMC2020496

[10] *International Burn Injury Database*. https://www.ibidb.org/ (cited July 2023).

[11] Soto C, Albornoz C, Peña V *et al.* Prognostic factors for amputation in severe burn patients. *Burns* 2013; **39**: 126–129. 22464750 10.1016/j.burns.2012.03.001

[12] Carrougher G, McMullen K, Mandell S *et al.* Impact of burn-related amputations on return to work: findings from the burn injury model system national database. *J Burn Care Res* 2019; **40**: 21–29. 30376104 10.1093/jbcr/iry057

[13] Bartley C, Atwell K, Purcell L *et al.* Amputation following burn injury. *J Burn Care Res* 2019; **40**: 430–436. 31225899 10.1093/jbcr/irz034 PMC6587426

[14] Sevil FC, Sevil H, Tort M, Öztürk M. Analysis of the efficacy of iloprost treatment in amputations due to burn. *J Burn Care Res* 2021; **42**: 82–86. 32735678 10.1093/jbcr/iraa121

[15] Wall S, Osman Y, Buthelezi X, Allorto N. Amputations secondary to burn injuries in a resource-limited setting. *Injury* 2022; **53**: 1716–1721. 34986979 10.1016/j.injury.2021.12.035 PMC9086096

[16] NHS England. *NHS standard contract for specialised burns care* (all ages). https://www.england.nhs.uk/wp-content/uploads/2014/04/d06-spec-burn-care-0414.pdf (cited December 2024).

[17] Stylianou N, Buchan I, Dunn KW. A review of the International Burn Injury Database (iBID) for England and Wales: descriptive analysis of burn injuries 2003–2011. *BMJ Open* 2015; **5**: e006184. 10.1136/bmjopen-2014-006184 PMC4346673 25724981

[18] Gurbuz K, Demir M, Basaran A, Das K. Most prominent factors contributing to burn injury-related amputations: an analysis of a referral burn center. *J Burn Care Res* 2021; **43**: 921–925. 10.1093/jbcr/irab219 34788839

[19] Tarim A, Ezer A. Electrical burn is still a major risk factor for amputations. *Burns* 2013; **39**: 354–357. 22853969 10.1016/j.burns.2012.06.012

[20] Harshman J, Roy M, Cartotto R. Emergency care of the burn patient before the burn center: a systematic review and meta-analysis. *J Burn Care Res* 2019; **40**: 166–188. 30452685 10.1093/jbcr/iry060

[21] Lawrence E, Li F. Foot burns and diabetes: a retrospective study. *Burns Trauma* 2015; **3**: 24. 27574670 10.1186/s41038-015-0024-6 PMC4963922

[22] Iles KA, Heisler S, Chrisco L *et al.* In patients with lower extremity burns and osteomyelitis, diabetes mellitus increases amputation rate. *J Burn Care Res* 2021; **42**: 880–885. 10.1093/jbcr/irab093 34057999

[23] Perrault D, Cobert J, Gadiraju V *et al.* Foot burns in persons with diabetes: outcomes from the national trauma data bank. *J Burn Care Res* 2022; **43**: 541–547. 35395676 10.1093/jbcr/irac021

[24] Rotman S, Lapaine P, Rehou S *et al.* Comparison of clinical outcomes of lower extremity burns in diabetic and nondiabetic patients: A retrospective analysis. *J Burn Care Res* 2022; **43**: 93–97. 34329452 10.1093/jbcr/irab150

[25] Adigun I, Ogundipe K, Abiola O. Amputation from burn following epileptic seizure. *Surg J* 2008; **3**: 78–81.

[26] Li Q, Wang L, Chen Q *et al.* Amputations in the burn unit: a retrospective analysis of 82 patients across 12 years. *Burns* 2017; **43**: 1449–1455. 28778757 10.1016/j.burns.2017.04.005

[27] Özalp B, Calavul A. Amputations in burn patients with a special emphasis on pediatric patients. *Erciyes Med J* 2017; **39**: 7–11.

[28] Schulze SM, Weeks D, Choo J *et al.* Amputation following hand escharotomy in patients with burn injury. *Eplasty* 2016; **16**: e13. 26977219 PMC4780278

[29] Haberal M, Sakallioglu Abali AE, Karakayali H. Fluid management in major burn injuries. *Indian J Plast Surg* 2010; **43**: S29–S36. 21321653 10.4103/0970-0358.70715 PMC3038406

[30] Cancio LC, Jimenez-Reyna JF, Barillo DJ *et al.* One hundred ninety-five cases of high-voltage electric injury. *J Burn Care Rehabil* 2005; **26**: 331–340. 16006840 10.1097/01.bcr.0000169893.25351.a9

[31] Hsueh Y, Chen C, Pan S. Analysis of factors influencing limb amputation in high-voltage electrically injured patients. *Burns* 2011; **37**: 673–677. 21334820 10.1016/j.burns.2011.01.014

[32] Jang K, Joo S, Jo J, Seo C. Burn and amputations: a retrospective analysis 379 amputation out of 19,958 burns in 10-year. *Int J Phys Med Rehabil* 2018; **6**: S148.

[33] Dash S, Arumugam PK, Muthukumar V *et al.* Study of clinical pattern of limb loss in electrical burn injuries. *Injury* 2021; **52**: 1925–1933. 33902868 10.1016/j.injury.2021.04.028

[34] Harish V, Tiwari N, Fisher OM *et al.* First aid improves clinical outcomes in burn injuries: evidence from a cohort study of 4918 patients. *Burns* 2019; **45**: 433–439. 30337155 10.1016/j.burns.2018.09.024

